# A Study of the Microstructure and Tribological Properties of Copper-Based Cr@graphite Alloy Modified by Nano Cr_3_C_2_ and CrC

**DOI:** 10.3390/nano13162347

**Published:** 2023-08-15

**Authors:** Yiran Wang, Yimin Gao, Yefei Li

**Affiliations:** State Key Laboratory for Mechanical Behaviour of Materials, School of Materials Science and Engineering, Xi’an Jiaotong University, Xi’an 710049, China

**Keywords:** nano Cr_2_O_3_, CrC, tribological property, copper-based graphite alloy

## Abstract

Railway switch plates are important components in railroads, and copper-based graphite alloys have potential as substitutes for traditional materials. Graphite as an anti-friction phase could decrease both the friction coefficient and mechanical properties, with an increasing mass fraction for their poor interface bonding strength. Chromium, a multifunctional metal plated on the graphite (Cr@graphite), has solved this problem. Results have shown that a copper-based Cr@graphite alloy is composed of copper as a base, and graphite and Cr compound transition phase as reinforcements. The transition phase is made up of nano Cr_3_C_2_ and dispersed CrC, which offers a stable combination with both graphite and copper. The tribological property of copper-based graphite alloy exhibits a steadily decreasing slope with reinforcement content increasing, and the Cr@graphite samples show lower values than the alloy without any coating treatment. Both graphite and chromic oxide play role in antifriction, and are more efficient than graphite alone. Microcutting is the dominant wear method when copper-based Cr@graphite alloy has a 1~4 wt.% reinforcements content; additionally, adhesion wear and oxidation are also generated. When the anti-friction phase increases, the wear mechanism is affected, and fatigue deformation is the dominant wear method at 4~6 wt.% content. The formation of the chromic oxide phase, as well as the graphite phase, control the formation of an anti-friction layer. In that case, the tribological properties are dramatically improved with reinforcement content enhance.

## 1. Introduction

Railway switch plates are important components for railroads, and electroless Cr-coated Q235 steel is in service as a current material. In the future, it will be necessary to develop new materials with self-lubrication, low friction coefficients, excellent wear resistance and anticorrosion performance to meet the demands of development [1]. Many studies have concentrated on self-lubrication materials for the railroads; typically, Bishop et al. conducted tests for self-lubrication using different self-lubricating composites to find alternative materials [2]. Liu et al. studied an oil lubricated resin with good anti-friction performance, and its friction coefficient was only 0.15 [3]. Li et al. investigated a self-lubrication epoxy as a coating for railroads [4]. They concluded that the antifriction mechanism of graphite and MoS_2_ was beneficial. Consequently, graphite can effectively modify the wear resistance property of the epoxy composite. Furthermore, it is also noted that the West Japan Railway Company and the Kyushu Railway Company in Japan developed oil-lubricated ceramics and alloys, respectively. The British railway also studied a new self-lubricating polymer for the railroads [5].

Copper-based graphite alloys, with their advantages of excellent tribological properties, good corrosion resistance and low costs, have shown potential as substitutes for traditional materials [6,7,8]. Nevertheless, graphite as a lubricant could decrease both the friction coefficient and mechanical properties with increasing mass fraction [9]. In our previous studies, this is because their exhibited interface bonding strength was very low [10]. To solve this problem, plating different metals on the graphite was adopted, the most-studied metals are Ni [11] and Cu [12]. In addition, new metals were investigated to improve both interface bonding and tribological properties, such as Ti [13], Sn [14] and Ag [15]. A multifunctional metal plated on the graphite can be beneficial for both mechanical and tribological properties, especially the self-lubrication performance produced by plated metal (or its in situ compound). Chromic oxide, another antifriction phase, also shows good tribological properties that can form self-lubrication performance.

To date, few researchers have focused on preparing Cr@graphite and utilizing chromium carbide to enhance the interface bonding strength of copper-based Cr@graphite alloy. Existing literature has shown it is difficult to prepare Cr@graphite through the traditional electroplating process. In recent years, multiple-arc ion plating has been used as a physical vapor deposition technology for depositing thin films on silicon steel sheets, but no one has employed this technology to plate particles or even graphite [16,17]. Nevertheless, coating Cr and its compound onto the graphite by multiple-arc ion plating has achieved success in this study.

This paper describes the effect of nano Cr_3_C_2_ and CrC on mechanical and tribological properties of a copper-based Cr@graphite alloy. The purpose of the present work is twofold. Firstly, the interface and mechanical property are improved by plating Cr and its compound onto the graphite. Cr was selected for coating because of its strong carbide-forming property. In addition, chromium carbide was reacted in situ as a transition phase due to its strong cohesion with both graphite and copper. The second purpose is to understand the tribological property of the copper-based Cr@graphite alloy. In particular, the self-lubrication performance is investigated by analyzing the chromic oxide, which is the main friction reaction product on the wear surface.

## 2. Experiments

### 2.1. Preparation of Cr@graphite

**Figure 1:**
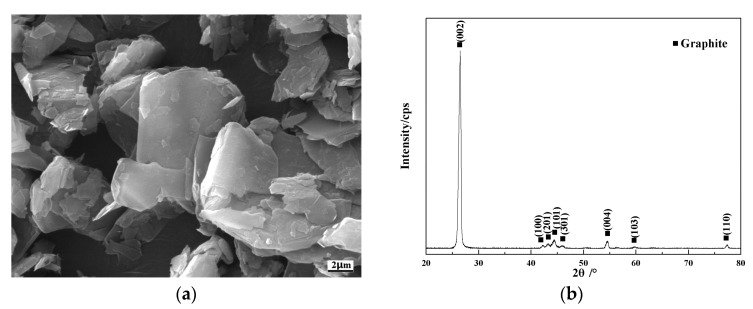
Microstructure and XRD results of flake graphite. (**a**) Morphology of flack graphite, (**b**) XRD results of flake graphite.

Cr@graphite particles were made of flake graphite powder. The microstructure of graphite is illustrated in Figure 1a. The size of the raw graphite is 325 mesh. Detailed properties of the raw graphite are shown in Table 1. The XRD results is illustrated in Figure 1b.

**Figure 2:**
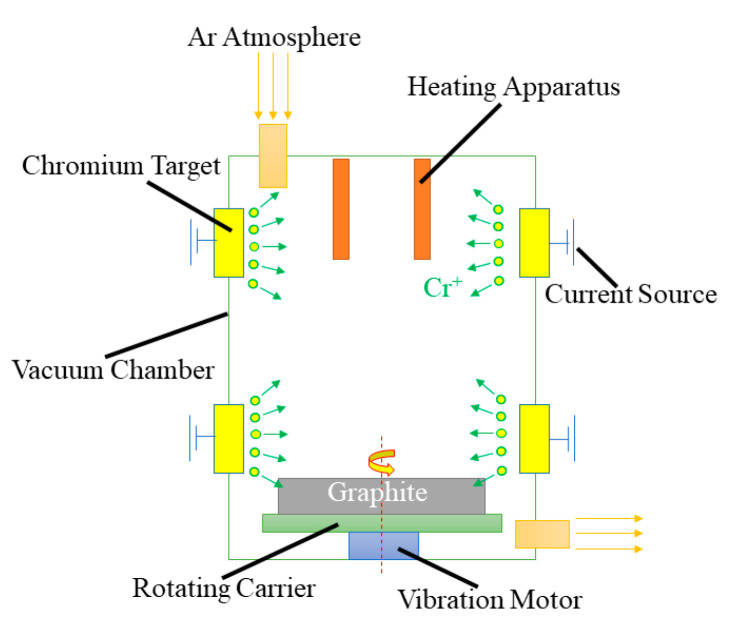
The schematic diagram of multiple-arc ion plating.

In the plating process, multiple-arc ion plating was adopted to prepare Cr@graphite. A schematic of multiple-arc ion plating is illustrated in Figure 2, and the composition of Cr target (99.98% purity) is shown in Table 2. The specimens were located in a rotating carrier on the bottom. Chromium targets were used to plate Cr ions through a multiple-arc process. The plating process was held at 573 K under 0.04 Pa argon atmosphere for 60 min. The duty cycle was 30%, and the bias voltage was 80 V.

### 2.2. Copper-Based Cr@graphite Alloy

The compositions of copper-based Cr@graphite alloy with 1~6% graphite content are given in Table 3. Copper powder (99.9% purity) with a 37.5 µm grain size and nickel powder (99.9% purity) with a 45 µm grain size were used for the alloy. The preparation of the alloy involved a powder metallurgical process and the whole process consisted of ball milling, compacting, sintering, re-compacting and re-sintering. The specific processing parameters are reported in Table 4. The size of the specimens was *Φ*44 × 6 mm.

### 2.3. Microstructure Investigation

Microstructure and phase analyses of the alloy were conducted by scanning electron microscopy (SEM), energy dispersive spectroscopy (EDS) and XRD detector (PANalytical X’PERTMPD), with an angular 2θ range from 20° to 80° and a step size of 0.01°. Both SEM and high-resolution transmission electron microscopy (HRTEM) were used to investigate the interface characterization. HRTEM was carried out by a 400 kV transmission electron microscope.

### 2.4. Mechanical Properties Test

In this study, mechanical property tests mainly included relative density, Vickers hardness and bending strength. The relative density was measured by Archimedes’ principle. Vickers hardness meters were used to test the bulk samples with a dwell time of 15 s and a load of 5 N. The bending strength of the alloy was determined by using three-point tests in a universal testing machine, whose specimens were cut into 28 × 5 × 5 mm bars. The results were calculated using the following Equation (1):

(1) $$\sigma_{f}=\frac{3PL}{2bd^{2}}$$

where *P* is the maximum load at fracture, *L* is the span length, and *b* is the width of and *t* is the thickness of the samples. After the tests, fracture morphology was observed by SEM.

### 2.5. Tribological Properties Test

A pin-on-disk contact geometry utilized rotatory sliding wear test was conducted in this study. Copper-based Cr@graphite alloys were slid against U75V steel with different reinforcement contents. U75V steel is a typical rail steel with single-phase pearlite, whose composition consisted of 0.77 wt.% C, 0.71 wt.% Si, 0.82 wt.% Mn, 0.041 wt.% V. The alloys were disks with a size of *Φ*44 mm × 5 mm at the bottom, and the U75V steel was pinned with a size of 5 × 5 × 20 mm at the top, in which one side was ground to a *Φ*5 mm half-spherical surface. Each experimental run took 1530 s at a speed of 0.065 m/s, a load of 20 N, and the test was conducted without lubricant at an average temperature of approximately 293 K. All wear tests were conducted five times under the same conditions. The alloy was weighed in each test, then, the relation Equation (2) was used to calculate the wear rate.

(2) $$\Delta V=\frac{\left(W_{1}-W_{2}\right)}{\rho \cdot L\cdot A}$$

where Δ*V* is the wear rate, *W*_1_ is the weight of the disk before the test, *W*_2_ is the weight of the disk after the test, *ρ* is the density of the disk, *L* is the sliding distance, and *A* is the contact area.

The microstructure of the worn alloy was observed by SEM and a 3D laser scanning profilometer. X-ray photoelectron spectroscopy (XPS) was conducted to study the wear product. All wear debris was collected and observed by SEM.

## 3. Results

### 3.1. Microstructure of Cr@graphite

**Figure 3:**
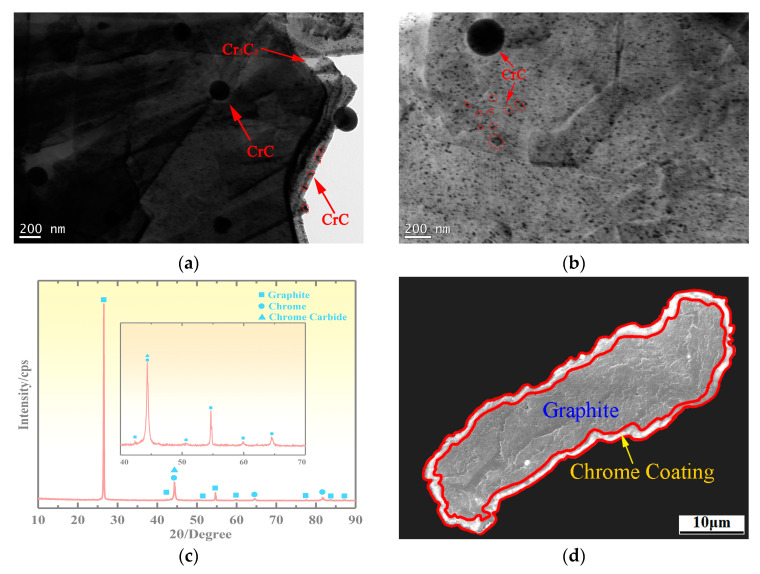

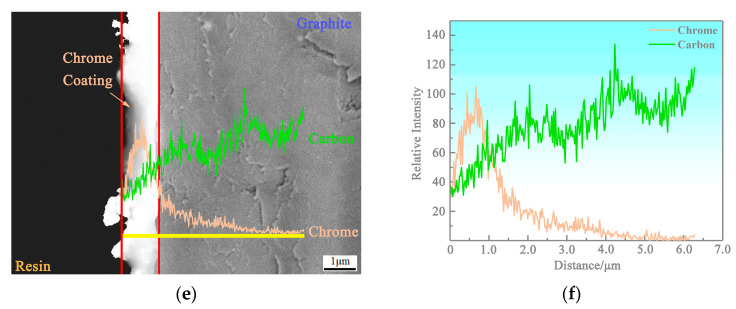
Microstructure characteristics of the Cr@graphite. (**a**,**b**) TEM Morphology of Cr@graphite, (**c**) XRD results of Cr@graphite, (**d**) cross-section morphology of Cr@graphite, (**e**,**f**) the linear scanning for interface of the coating and graphite.

The microstructure of the Cr@graphite is shown in Figure 3. There is an obvious shell layer on the surface of Cr@graphite, and a large number of white spherical particles with an average size of 2.18 μm are dispersed. XRD analysis is conducted on the Cr@graphite, and the results are shown in Figure 3c. It is shown that the Cr@graphite consists of graphite and nano Cr_3_C_2_ and CrC. The cross-section of the graphite and the interface between the coating and graphite are analyzed by SEM and EDS, and the analysis results are shown in Figure 3d–f. The graphite particles are coated with a Cr_3_C_2_ layer, and the coating is uniform and dense, without obvious holes and porosity. The Cr_3_C_2_ layer is well-combined with graphite, and the thickness of the Cr_3_C_2_ is approximately 1.12 μm. The linear scanning results of Figure 3f show that C atoms and Cr atoms diffuse smoothly in the Cr_3_C_2_ coating and graphite without reaction. The diffusion of C atoms in the Cr layer is obvious, and the diffusion amount is very stable and does not decrease with increasing diffusion distance. Line scanning analysis indicates that the distribution of Cr in the coating is parabolic. The Cr atom diffuses to the Cr–C interface. During the plating process, the atom transfers from the outside to the inside of the coating. The formation of Cr_3_C_2_ prevents the diffusion of C atoms. The content of C atoms in the Cr coating is lower, and the diffusion amount decreases with increasing diffusion distance.

Previous research [18] on the relationship between the coating time and the surface of Cr@graphite has shown that, in the early stage of coating, the diffusion of Cr and C occurs under the bombardment of multiple-arc ion plating, and nano H-Cr_7_C_3_ is formed by the reaction. With the progress of the coating process, the diffusion of Cr and C becomes more sufficient, and the ion bombardment continuously generates energy so that the coating material H-Cr_7_C_3_ is completely transformed into nano Cr_3_C_2_, forming the Cr_3_C_2_ shell layer. At the same time, the diffusion of Cr and C atoms causes Cr_3_C_2_ to undergo phase transformation and react to form nanosized CrC. CrC has no orientation relationship with Cr_3_C_2_, and there is an obvious overplanting layer in the middle. The spherical particles are CrC. The graphite surface is composed of spherical particles, nano dispersants, and coatings. The nano dispersant particles are crystallized and have not yet grown, and the average size is approximately 180 nm.

### 3.2. Microstructure of the Copper-Based Cr@graphite Alloy

**Figure 4:**
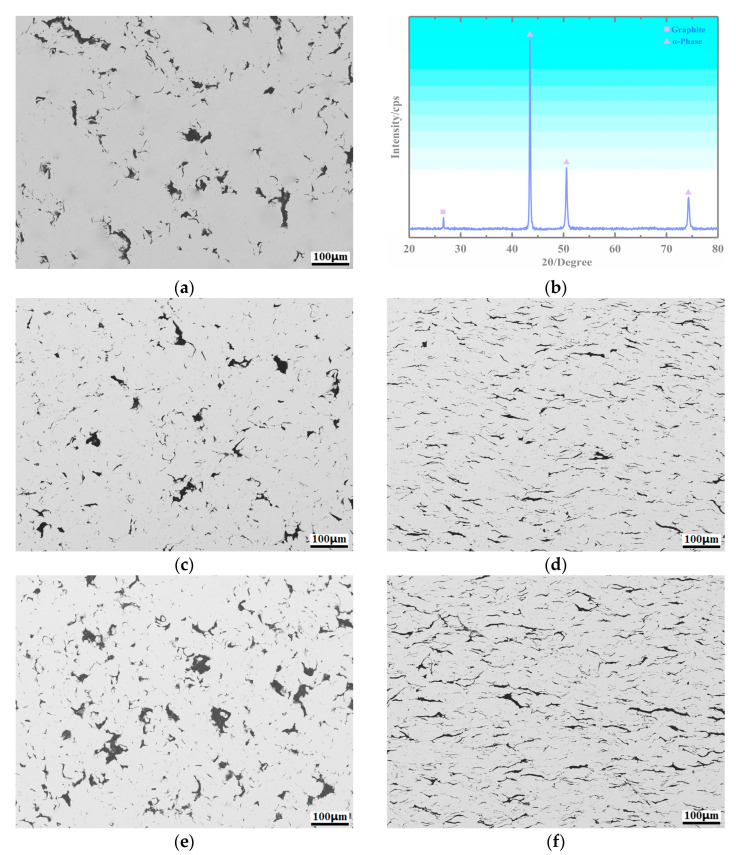
Microstructure of copper-based Cr@graphite alloy. (**a**) Surface morphology of Cr2, (**b**) XRD results, (**c**) surface morphology of Cr4, (**d**) cross-section morphology of Cr4, (**e**) surface morphology of Cr6, (**f**) cross-section morphology of Cr6.

After multiple-arc ion plating, Cr@graphite and copper are prepared for copper-based Cr@graphite alloy by powder metallurgy. The XRD results show in Figure 4b that the alloy has two phases: a matrix in the grey zone (copper) and graphite in the black zone. Graphite shows a distribution from dispersed to a semicontinuous net when the reinforcement increased. As the raw material used in this study, flake graphite shows excellent lubrication properties, with low strength and high aspect ratio. Therefore, the microstructure of the alloy has a great relationship with the direction of the cold pressing. The morphology has two types: surface morphology in the direction of vertical pressing and cross-section morphology in the direction of parallel pressing, and the results are shown in Figure 4. The graphite in the direction of vertical pressing exhibits a block shape, whereas the graphite becomes a linear sharp in the direction of parallel pressing.

**Figure 5:**
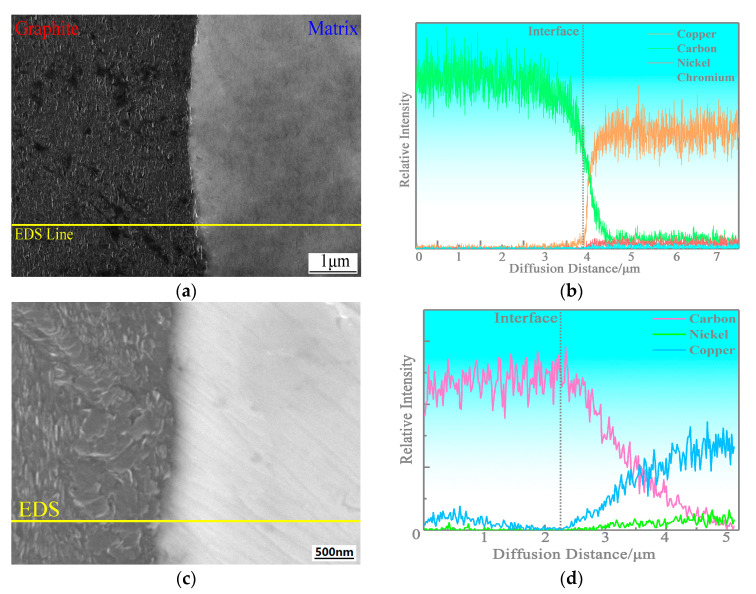
The interfacial morphologies of copper-based Cr@graphite alloy and copper-based graphite alloy. (**a**) Interfacial morphologies of copper-based Cr@graphite alloy, (**b**) interfacial morphologies of copper-based graphite alloy, (**c**) EDS results of copper-based Cr@graphite alloy, (**d**) EDS results of copper-based graphite alloy.

The interface morphologies of copper-based Cr@graphite alloy are expressed in Figure 5, whereas the EDS results also show quite different trends. It is difficult to find a transition phase at the interface in the alloy; however, its EDS results show that the C element diffuses from the graphite to the copper by only 0.48 μm, and the Cu element diffuses approximately 0.26 μm. Then, 0.58 μm width mutual diffusion has emerged. As a comparison, for the alloy without any metal coating treatment, only the C element diffuses 1.92 μm, and no mutual diffusion is emerged. Therefore, only a single-track diffusion occurs at the interface in the unmodified sample.

### 3.3. Mechanical Properties Results

**Figure 6:**
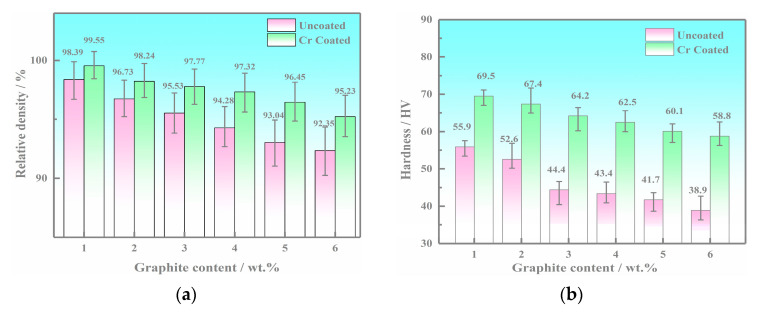

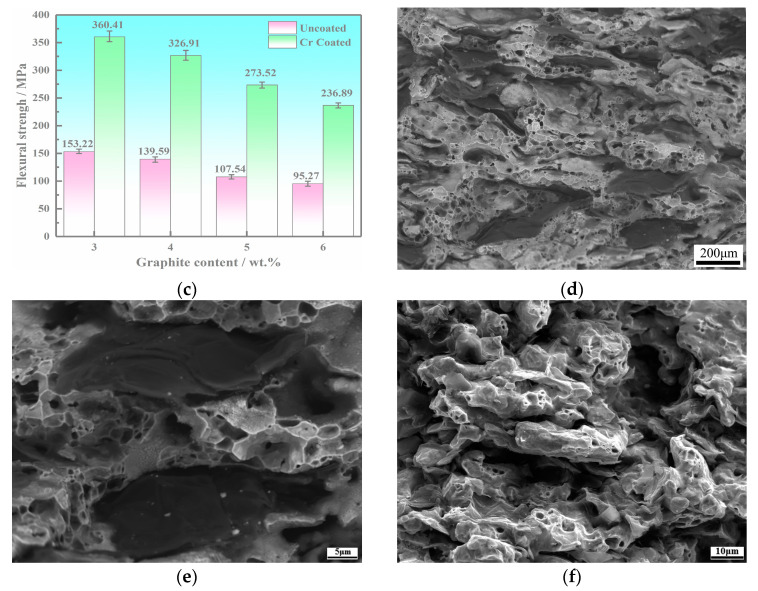
Mechanical properties results of the copper-based Cr@graphite alloy. (**a**) Relative density, (**b**) Vickers hardness, (**c**) bending strength, (**d**) fracture morphologies of Cr4 at low magnification, (**e**) fracture morphologies of Cr4 at high magnification, (**f**) fracture morphologies of the alloy without coating treatment.

The relative density, Vickers hardness, and bending strength of copper-based Cr@graphite alloy are illustrated in Figure 6. The variation trend of the three properties exhibits a monotonic decrease with increasing graphite content. The values of the alloy with Cr coating treatment are much higher than those of the alloy without any coating treatment under the same reinforcement contents. It can be concluded that the relative density, Vickers hardness, and bending strength increase by 25.16%, 39.39%, 143.10%, respectively, by the formation of the nano Cr_3_C_2_ and CrC phase. It can be explained by the interface changing from machinal bonding to metallurgical bonding, and the improvement in interface bonding strength significantly reflects the increase in mechanical properties.

The fracture morphologies are shown in Figure 6d–f; many dimples can be seen, and the failure mode of the alloy is ductile fracture. It can be summarized that the fracture of the graphite is a transgranular fracture, and there are no spalling pits. However, the fracture mechanism of the alloy without coating treatment is dimples and an intergranular fracture, in which the graphite is spalled out of the alloy. Therefore, it can be concluded that the nano Cr_3_C_2_ and CrC could prevent cracks from spreading along the interface by the generation of chromium compounds. Moreover, solid solution strengthening also emerges through Cr element diffusion into the copper. In conclusion, the copper-based Cr@graphite alloy is strengthened by transformation of the interface bonding method and solid solution strengthening.

### 3.4. Wear Test Results

**Figure 7:**
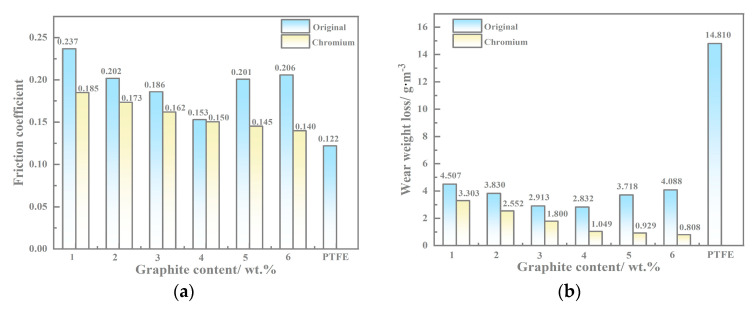
Tribological properties results of the copper-based Cr@graphite alloy. (**a**) Friction coefficients, (**b**) wear rate.

The friction coefficients of the copper-based Cr@graphite alloy and the comparison specimen (the alloy without coating treatment and PTFE) are shown in Figure 7a. It is notable that the alloy with Cr coating treatment shows lower values than the alloy without coating treatment. As discussed in a previous study, the friction coefficient of the alloy without coating treatment tends to descend from 1 wt.% to 4 wt.% graphite content, and rises from 4 wt.% to 6 wt.% due to the delamination and fatigue wear caused by the weak interface bonding when the content exceeds 4 wt.%. Nevertheless, the friction coefficients of the copper-based Cr@graphite alloy exhibit a steady descent slope with increasing content. It seems that nano Cr_3_C_2_ and CrC plays an essential role in the antifriction performance, as well as restraint delamination and fatigue generation when the content enhances.

The wear rate of the copper-based Cr@graphite alloy is illustrated in Figure 7b. The values of the alloy with the nano Cr_3_C_2_ and CrC phase are much lower than the others, especially at high contents. When the content is more than 4 wt.%, the wear rate is deeply affected by interface bonding, and the Cr coating treatment method shows remarkable promotion. Consequently, the nano Cr_3_C_2_ and CrC form antifriction performance during the sliding, which could improve both the wear rate and friction coefficients. As another potential material, PTFE’s friction coefficient is slightly lower than that of copper-based Cr@graphite alloy; however, the wear rate presents a much higher value.

## 4. Discussion

### 4.1. Interfacial Improvement of Copper-Based Cr@graphite Alloy

**Figure 8:**
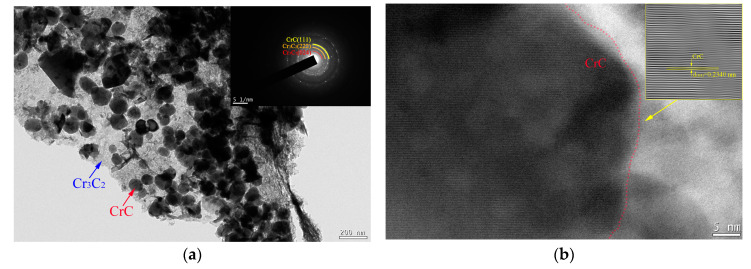

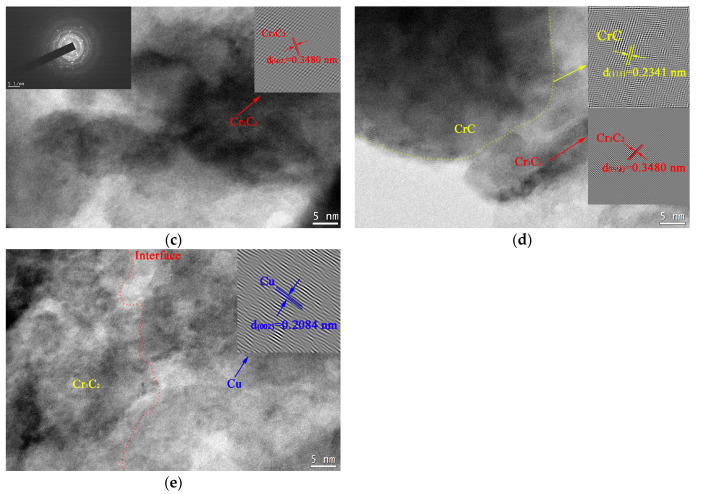
TEM morphology of the interface in the copper-based Cr@graphite alloy. (**a**) Microstructure of the interface, (**b**) HRTEM morphology of nano CrC, (**c**) HRTEM morphology of nano Cr_3_C_2_, (**d**) interfacial morphology of nano Cr_3_C_2_ and CrC, (**e**) interfacial morphology of Cr_3_C_2_ and Cu.

A TEM investigation is conducted to further analyze the microstructure of the interface, and the morphologies are illustrated in Figure 8. This result demonstrates that the transfer phase between graphite and the copper at the interface is composed of many fine nanoparticles and their intermediate phase. According to the diffraction spot from the transfer phase, it can be proven that this interfacial area consists of nano Cr_3_C_2_ and CrC. The high-resolution microstructure image of the fine nanoparticles is displayed in Figure 8b; it is noted that the particle is confirmed as the nano CrC phase. With the same shape and preferred growth plane, the CrC exhibits a spherical shape and preferred growth at the (111) lattice plane. Then, the intermediate phase is proven to be the nano Cr_3_C_2_ phase, and its TEM morphology is illustrated in Figure 8c. It is noted above that Cr@graphite consists of graphite and nano Cr_3_C_2_ and CrC; hence, the nano Cr_3_C_2_ evolves into the intermediate phase at the interface of the alloy.

It is noteworthy that the number of CrC nanoparticles at the interface is much greater than that of the Cr@graphite. The interface of nano Cr_3_C_2_ and CrC is illustrated in Figure 8d, which shows the reason that these CrC nanoparticles are generated. When the C element diffuses from graphite to copper, it should cross the nano Cr_3_C_2_ intermediate phase, and most of the carbon atoms react with Cr_3_C_2_ to form CrC. The reaction can be explained by Formula (3):

Cr_3_C_2_ + C → 3CrC (3)

Therefore, stable metallurgical bonding is formed by nano Cr_3_C_2_ and graphite, which is further supported by the fact that C element atoms diffuse from graphite to the copper by only 0.48 μm, according to the EDS lining. The interface of nano Cr_3_C_2_ and Cu is also analyzed, as shown in Figure 8e. It is noted that the orientation relationship between nano Cr_3_C_2_ and α-Cu is Cr_3_C_2_ (111)//Cu (002); therefore, the nano Cr_3_C_2_ phase also forms a good interface combination with the copper.

In summary, according to the alloy with Cr coating treatment, the interface is changed to metallurgical bonding. The transfer phase consists of nano Cr_3_C_2_ and dispersed nano CrC, which has a stable combination with both graphite and the matrix. In this case, the interface is dramatically strengthened; consequently, both the mechanical and tribological properties are improved.

### 4.2. Anti-Friction Performance of Cr_2_O_3_

**Figure 9:**
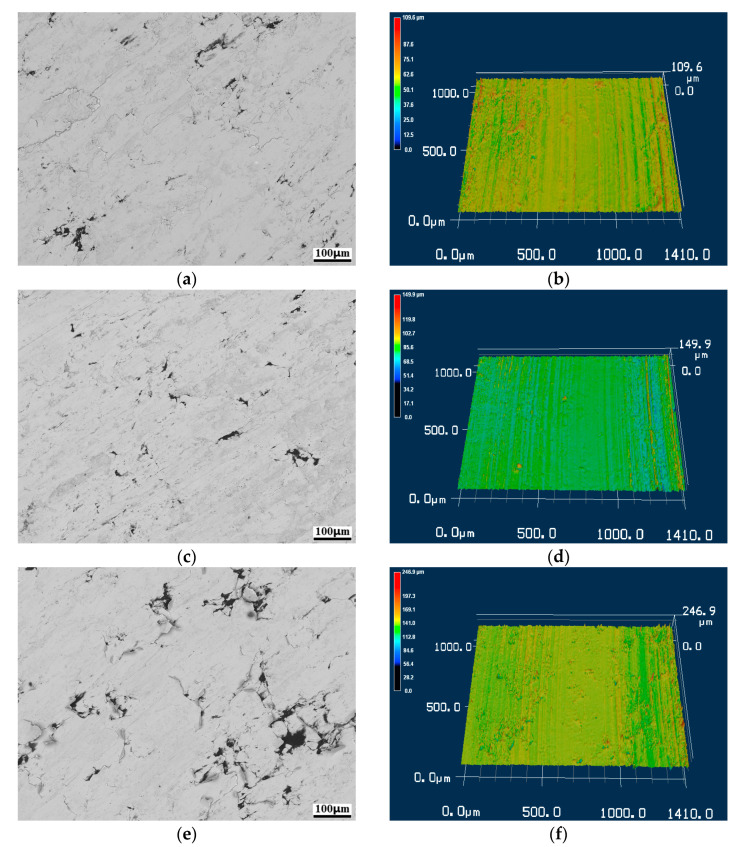
The morphologies of the worn surface. (**a**) C2 sample SEM image, (**b**) C2 sample 3D laser scanning image, (**c**) C4 sample SEM image, (**d**) C4 sample 3D laser scanning image, (**e**) C6 sample SEM image, (**f**) C6 sample 3D laser scanning image.

The morphologies of the worn surface are illustrated in Figure 9, where Figure 9a,c,e represent the copper-based Cr@graphite alloy with 2~6 wt.% reinforcement contents. It can be inferred that many fine furrows on the alloy, as well as fatigued laminate sheets, emerge in the Cr6 sample. The graphite shows a good combination with the matrix, and the lubrication film can also be observed on the worn surface. As a comparison, the unmodified composites show many spalling pits due to their poor interfacial bonding in previous studies. According to the 3D laser scanning results, as exhibited in Figure 9b,d,f, the furrows in the Cr4 sample are less deep than those in the Cr2 and Cr6 samples. This is because an anti-friction layer is generated on the alloy, and more reinforcement content introduces a stronger anti-friction performance. When the reinforcement content at 6 wt.%, fatigue wear affects the interfacial bonding intensity, and both furrows and fatigued laminate sheets are formed on the worn surface. Therefore, the surface roughness shows a tendency to decline and then rise.

**Figure 10:**
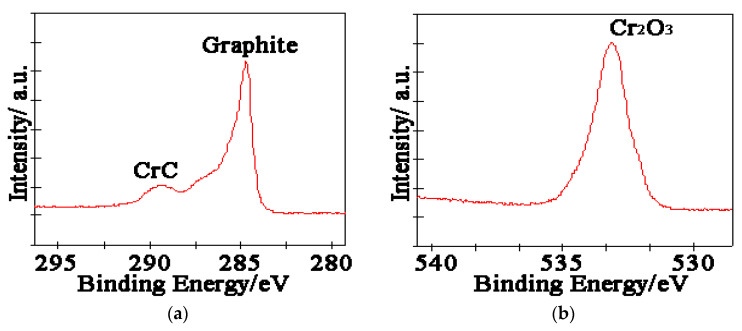
XPS results. (**a**) C_1s_, (**b**) O_1s_.

**Figure 11:**
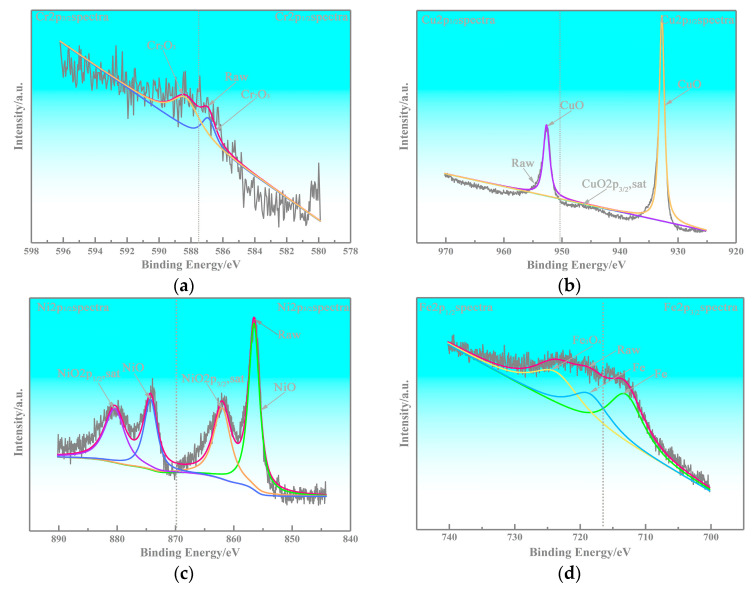
XPS results. (**a**) Cr_2p_, (**b**) Cu_2p_, (**c**) Ni_2p_, (**d**) Fe_2p_.

The worn products of the alloy are analyzed by XPS, and the results are given in Figure 10 and Figure 11. The results illustrate that Cr_2_O_3_ is present on the worn surface, which can play an essential role as a lubricant through shear deformation. Therefore, it can be inferred that the nano Cr_3_C_2_ and CrC phases at the interface can quickly generate oxidation during the sliding process. The reaction can be explained by Formulas (4) and (5):

4Cr_3_C_2_ + 9O_2_ → 6Cr_2_O_3_ + 8C (4)

4CrC + 3O_2_ → 2Cr_2_O_3_ + 4C (5)

Many studies have been published that assess the anti-friction performance of graphite, as a friction-reducing phase, can generate an anti-friction layer to modify the tribological properties [19,20,21]. Cr_2_O_3_ could also form anti-friction performance, and this trend is visible shown in Figure 7; the tribological properties of the copper-based Cr@graphite alloy exhibit higher performance than those of the alloy without any coating treatment. The Fe_2p_ spectra in Figure 10d exhibited that Fe and Fe_3_O_4_ transferred on the alloy, which induce the adhesive wear. In addition, CuO and NiO can confirm that the oxidation wear also occurs, whereas the oxidation wear does not seem severe because only Fe_3_O_4_ is generated, but not Fe_2_O_3_ or FeO. Therefore, the anti-friction layer is consists of graphite, Cr_2_O_3_, Cu, Fe, and other oxides.

**Figure 12:**
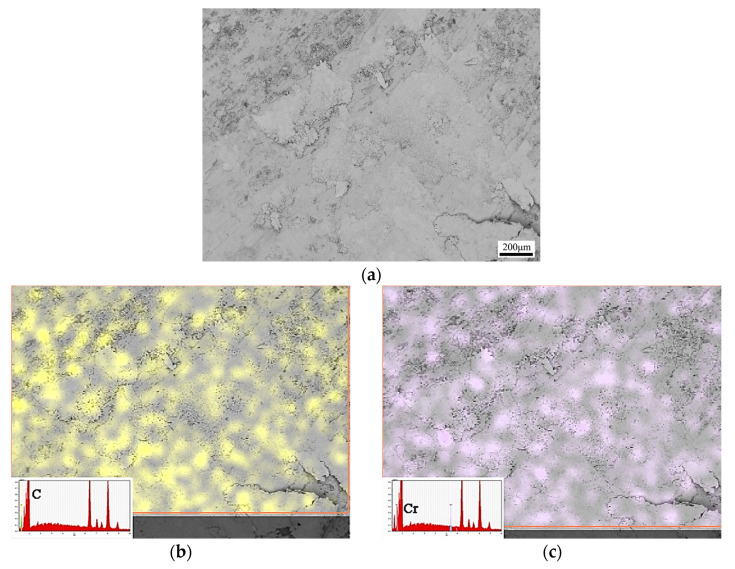

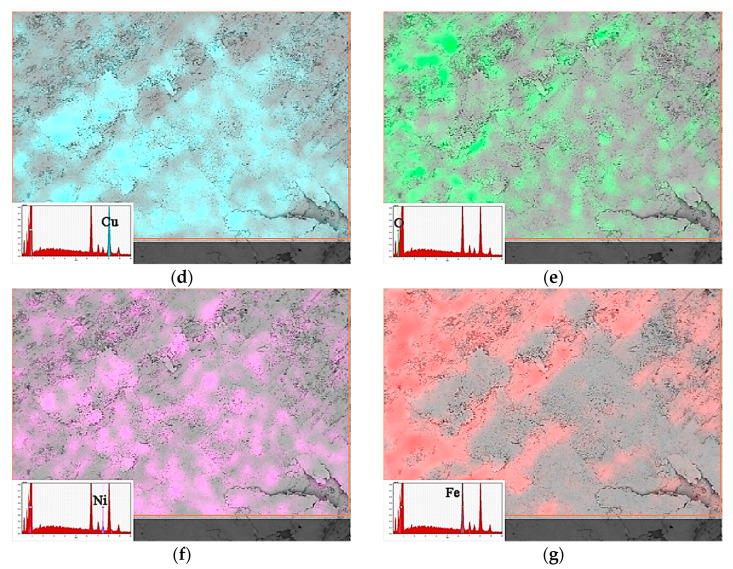
The morphology of the pins. (**a**) Wear morphology, (**b**) EDS results of C element, (**c**) Cr element, (**d**) Cu element, (**e**) O element, (**f**) Ni element, (**g**) Fe element.

The counterpart, U75V steel pins, whose morphology is illustrated in Figure 12a, are also studied. It can be inferred that the pin is attached to the wear product film, which is composed of brittle graphite, Cr_2_O_3_, Cu and other oxides. According to the EDS in Figure 12b–g, the results confirm that adhesive wear and mass transfer occur from the disk to the pin. The C element coming from the graphite is dispersed on the pin; moreover, the Cr and O elements prove that Cr_2_O_3_ disperses on the surface of the pin. This anti-friction layer, mainly composed of graphite and Cr_2_O_3_, could gradually transfer from “layer vs. pin” to “layer vs. layer”. The pins sliding against the alloy without any coating treatment was also studied in our previous research, and the results showed that the pin without any layer on it seems really rough. In this case, the friction coefficient and wear resistance are definitely much higher than those of the alloy with Cr coating treatment. Hence, Cr_2_O_3_, which is generated from the nano Cr_3_C_2_ and CrC phases at the interface, also facilitates the adhesion of graphite, and shows a better anti-friction performance.

### 4.3. Wear Mechanism

**Figure 13:**
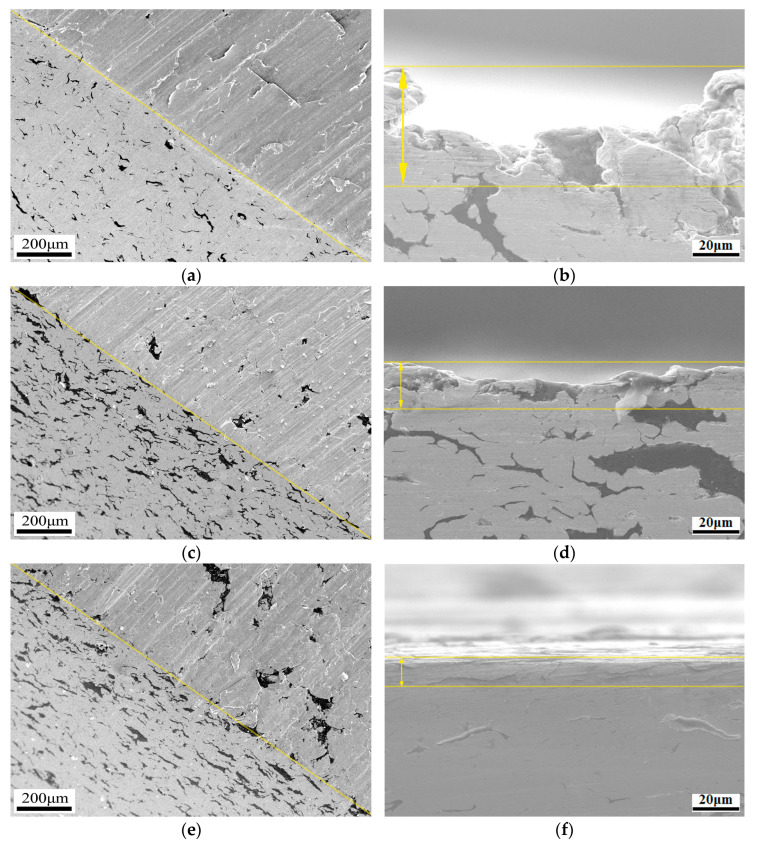
Diagonal plane section and cross-section morphologies. (**a**) Diagonal plane section of Cr2, (**b**) cross-section morphology of Cr2, (**c**) diagonal plane section of Cr4, (**d**) cross-section morphology of Cr4, (**e**) diagonal plane section of Cr6, (**f**) cross-section morphology of Cr6.

As discussed above, it can be inferred that microcutting is the main wear mechanism below 4 wt.% reinforcement content. With the content increasing, fatigue deformation gradually appears as another failure form. The cross-section and diagonal plane section are illustrated in Figure 13. It can be found that the depth of the furrows gradually reduces when the reinforcement content enhances, and the surface changes from rough to smooth, as shown in Figure 13a–c. As a comparison, the alloy without any coating treatment exhibits spalling pits and a rougher worn surface. Therefore, it can be concluded that graphite and Cr_2_O_3_ can restrict microcutting, especially at high graphite contents, and the furrows are much shallower, whereas the worn surface is almost straight, as shown in Figure 13d–f.

Fatigue deformation zones can also be investigated on the subsurface. From the morphologies, the fatigue deformation zone seems to gradually increase when the reinforcement content enhances. This is because Cr_2_O_3_ and graphite, which play a role in the anti-friction phase, can avoid microcutting failure and change the wear mechanism from microcutting at low graphite content to fatigue deformation at high graphite content. Therefore, both Cr_2_O_3_ and graphite can also ensure a low roughness surface and decrease the friction coefficients.

The microstructure of the cross-section in Figure 13f also proves that the change in the wear mechanism is induced by the increased lubrication generated. In other words, many cracks propagate on the subsurface of the alloy without any coating treatment when the reinforcement content exceeds 4 wt.%. Therefore, many spalling pits form and reduce the graphite lubrication. However, the chromium compound at the interface could not only prevent crack spreading, but also react to Cr_2_O_3_ as a lubricant. Consequently, spalling pits would not emerge in the alloy with Cr coating treatment at 6 wt.% graphite content.

**Figure 14:**
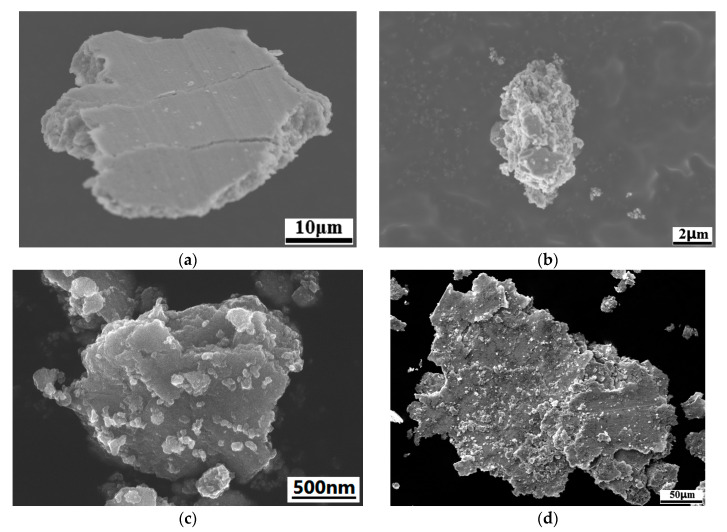
Morphologies of wear debris. (**a**) Cr2 sample, (**b**) Cr4 sample, (**c**) Cr6 sample, (**d**) the alloy without any coating treatment.

In addition, evidence of the wear debris of each alloy shown in Figure 14 also proves the mechanism evolution by the induction of the chromium compound. When the reinforcement content is 2 wt.%, the lubricating effect is relatively weak; therefore, microcutting is the main failure method and results in a large amount of wear debris. When the content increases, the microcutting is restricted by anti-friction performance; consequently, the amount of the wear debris is slight. However, the wear debris of the alloy without any coating treatment is more voluminous due to their severe rupture during sliding. Again, it is confirmed that the chromium carbide strengthens the interfacial bonding and decreases the friction coefficient, as well as the wear loss by the formation of Cr_2_O_3_.

According to the XPS results and the morphology of the pins, nonprimary wear mechanisms, such as oxidation and adhesion wear, are generated during sliding. In a previous study, this phenomenon usually emerged in the Cu-Fe wear system. In this study, these two wear mechanism are not the main failure methods in the different graphite content samples; however, oxidation and adhesion are essential for friction resistance enhancement. First, Cr_2_O_3_ is formed by the oxidation of nano Cr_3_C_2_ or CrC; in other words, the adhesion facilitates the lubrication film transformation to the pins (U75V steel) and ensures that the graphite and Cr_2_O_3_ distribute the whole worn surface of the disk and the pins.

As discussed above, the wear mechanism indicates that microcutting is the dominant wear method when the alloy below 4 wt.% reinforcement content; additionally, oxidation and adhesion wear are also generated. When the anti-friction phase increases, the wear mechanism is affected, and fatigue deformation is the dominant wear method at 4~6 wt.% graphite content. The formation of the Cr_2_O_3_ phase, as well as the graphite phase, controls the formation of the anti-friction layer. In that case, the tribological properties are dramatically improved when reinforcement content enhances.

## 5. Conclusions

(1) Cr@graphite, consisting of graphite and nano Cr_3_C_2_ and CrC, is successfully processed by multiple-arc ion plating. The copper-based Cr@graphite alloy is composed of graphite, copper, and a transition phase at the interface. The transfer phase is made up of nano Cr_3_C_2_ and dispersed CrC, which has a stable combination with graphite and copper. The interface bonding is changed to metallurgical bonding, resulting in the interface being dramatically strengthened, and the relative density, hardness, and flexural strength increase by 25.16%, 39.39%, and 143.10%, respectively;

(2) Tribological properties of the copper-based Cr@graphite alloy exhibit a steady descent slope with reinforcement content increasing, and the alloy with nano Cr_3_C_2_ shows lower values than the alloy without any coating treatment. The anti-friction phase comprises graphite, Cr_2_O_3_, Cu, Fe, and other oxide on the worn surface of both the disk and pin. Graphite and Cr_2_O_3_ could form a synergistic lubrication effect, and Cr_2_O_3_, which is generated from the nano Cr_3_C_2_ and CrC phases at the interface, facilitates anti-friction performance;

(3) The wear mechanism indicates that microcutting is the dominant wear method when 1~4 wt.% reinforcement content; additionally, oxidation and adhesion wear are also generated. When the anti-friction phase increases, the wear mechanism is affected, and fatigue deformation is the dominant wear method at 4~6 wt.% content. The formation of the Cr_2_O_3_ phase, as well as graphite phase, control the formation of the anti-friction layer. In that case, the tribological properties are dramatically improved when reinforcement content enhances.

## Figures and Tables

**Table 1:** Physical properties of flake graphite.

Properties	Density/g·m^3^	Purity /%	Melt Point /K	Specific Heat Capacity/J·(kg·K)^−1^	Conductivity /m·Ω	Thermal Conductivity/W·(m·K)^−1^
Parameters	2.25	99.5	4123 K	710	0.061 × 10^−6^	129

**Table 2:** Composition of Cr target.

Element	Cr	C	Si	Fe	P	S
Weight/%	99.9	0.008	0.09	0.17	0.005	0.003

**Table 3:** Compositions of copper-based Cr@graphite alloy.

	Contents	Cr@graphite/wt.%	Cu/wt.%	Ni/wt.%
Samples				
Cr1		1	91	8
Cr2		2	90	
Cr3		3	89	
Cr4		4	88	
Cr5		5	87	
Cr6		6	86	

**Table 4:** Specific processing parameters of copper-based Cr@graphite alloy.

Sequence of Steps	Processing	Parameters
1	Ball-milling	200 rpm × 15 h
2	Cold Compacting	600 MPa × 3 min
3	Sintering	1123 k × 1.5 h at vacuum
4	Re-Compacting	600 MPa × 3 min
5	Re-Sintering	1073 k × 0.5 h at vacuum

## Data Availability

The data used to support the findings of this study are available from the corresponding author upon request.
